# Shifting Perceptions and Emotional Responses to Autonomous Vehicles Using Simulated Experiences

**DOI:** 10.3390/bs14010029

**Published:** 2023-12-30

**Authors:** Jose L. Tapia, David Sánchez-Borda, Carmen Iniesta, Francisco Badea, Jon Andoni Duñabeitia

**Affiliations:** 1 Centro de Investigación Nebrija en Cognición (CINC), Universidad Nebrija, 28043 Madrid, Spain; jtapia@nebrija.es (J.L.T.); dsanchezb2@alumnos.nebrija.es (D.S.-B.); 2 Escuela Politécnica Superior, Universidad Nebrija, 28015 Madrid, Spain; carmen.iniesta@ufv.es (C.I.); fbadea@nebrija.es (F.B.); 3 Escuela Politécnica Superior, Universidad Francisco de Vitoria, 28223 Madrid, Spain

**Keywords:** autonomous vehicles, public acceptance, lexical selection task, emotional lexicon, demographic influences

## Abstract

The societal integration of autonomous vehicles (AVs) relies on public acceptance, closely related to individual emotions and perceptions. This study explores the emotional factors affecting AV acceptance in Spain through lexical tasks, virtual AV simulations, and questionnaires, surpassing traditional attitude surveys by examining subtle emotional and lexical reactions to AVs. Acceptance was measured in terms of AV knowledge, perception of autonomous driving, and safety, with emphasis on lexical-emotional analysis after simulation. Findings indicate gender differences in AV acceptance, with women showing less knowledge and comfort with AV technology. Simulation improved understanding and generated more positive responses. This study shows how lexical tasks can reveal emotional influences on AV perception and suggests a wider approach to assess technology acceptance. These findings aid in creating campaigns and experiences to enhance public AV acceptance, mindful of demographic differences. Future studies should extend this framework to various populations to investigate the emotional lexicon’s role in AV acceptance.

## 1. Introduction

Over the past decade, the field of artificial intelligence (AI) has notably pivoted towards the development of autonomous vehicles (AVs) [1]. Market projections suggest that by 2030, AVs could constitute as much as 5% of the global automobile market [2]. The potential widespread adoption of AVs promises transformative effects on society, especially in enhancing mobility and road safety [3,4,5,6,7]. Yet, these advantages are tempered by challenges that extend beyond mere technological hurdles. The successful integration of AVs into society and the driving culture demands the alignment of regulatory frameworks, social norms, and human behavioral factors [8,9]. As succinctly noted by Golbabaei et al. [10], the realization of these benefits is intrinsically tied to societal acceptance. In this context, psychological aspects like public attitudes, trust, and emotional reactions might pose as formidable, if not more significant, barriers to AVs adoption than any technological constraints. Despite broad public awareness, persistent apprehensions about safety and reliability remain considerable impediments [9,11]. Therefore, fostering greater user acceptance of autonomous driving technology emerges as a critical challenge.

Building on this premise, among the many factors influencing public acceptance of technology, behavioral elements like trust, perceived risk, and perceived safety stand out as particularly significant. Trust is strongly associated with the user’s past experiences, thus modulating user perceptions [12]. Therefore, initial impressions and experiences with the vehicle play an important role in shaping user confidence. Furthermore, emotions associated with vehicle operation are intrinsically linked to user trust [13]. Further complementing the notion of trust, perceived safety, defined as an environment where drivers and passengers feel at ease, safe, and comfortable while in transit [14], has been established as a reliable predictor of intentions to utilize AVs [15,16,17,18,19]. Concerns about operational safety and personal safety while using AVs have been well-documented [20,21,22], suggesting that safety could be a solid determinant in the public’s acceptance or rejection of AVs. Perceived safety encapsulates both emotional and cognitive elements, which have been linked to risk perception and technological acceptance [16,23]. From a cognitive perspective, predictability and a sense of control are deemed key to perceived safety, guiding individuals in making safe and reliable decisions while driving [24]. Conversely, the emotional dimension of safety is closely tied to establishing a sense of ease and comfort for passengers [25].

### 1.1. Background

While these behavioral components provide a foundational understanding, it is the interplay of emotions with these factors that further nuances our grasp on user acceptance. Extensive research has been conducted on how affect, understood as emotions, feelings, and affective states, is implicated in risk perception and decision-making processes. Central to this discourse are dual-process theories, which emphasize the role of intuitive and experiential processes in human decision-making [26,27]. The Somatic Marker Hypothesis posits that experiences automatically trigger emotional reactions that “mark” decision-making options, thereby facilitating the selection of advantageous choices, reducing the decision-making space, and enhancing the efficacy of reasoning [28]. For instance, if someone had a comfortable and safe ride in an AV, they may associate positive emotions with the technology, making them more inclined to choose an AV for future journeys. Furthermore, the Affect Heuristic demonstrates how reliance on integral emotions can simplify complex decisions [29]. Consider a scenario where a person needs to decide between driving themselves and using an autonomous vehicle during a snowstorm. Their reliance on the emotion of fear or anxiety, driven by the perceived risk of accidents in snowy conditions, might lead them to opt for the AV as a safer choice. Hence, in situations of uncertainty, decision-makers label options with either positive or negative affective impressions when rendering judgments. These intuitive emotions act as cognitive shortcuts, wherein positive affect decreases risk perceptions and negative affect increases them [30,31,32]. For example, hearing about a recent accident involving an AV may trigger negative emotions in an individual, leading to a perception of higher risks associated with AVs, even if statistically, the technology is safer than human-driven vehicles. This highlights the significance of emotional reactions in individuals’ choices, which may not always align with objective risk assessments.

Traditionally, studies on acceptance and perception have predominantly employed qualitative methods such as questionnaires and telephone interviews [33,34]. While these methods have proven to be valuable in gathering explicit attitudes and perceptions, they may fall short in capturing the emotional components that shape technology perceptions. Given the complex role that emotional variables play in influencing people’s attitudes towards AVs, a more comprehensive approach that focuses on subtle emotional responses is needed. In this light, utilizing lexical selection tasks emerges as a potentially more comprehensive method to explore these emotional underpinnings [35,36,37,38]. Lexical selection tasks offer a unique advantage as they allow for the exploration of emotional dimensions like valence and arousal in a manner that is less overt compared to traditional methods. Unlike questionnaires or interviews, where participants are explicitly asked about their feelings or attitudes, lexical selection tasks present as mere linguistic exercises to the participants. This subtlety is important as it keeps participants blind to the fact that their emotions are being evaluated, thereby reducing the likelihood of response bias that often accompanies self-reported measures. Participants are less likely to modify their responses to fit social desirability norms, hence providing a more authentic insight into their emotional states. Furthermore, the language individuals use inherently carries emotional connotations, which when analyzed, can reveal a wealth of information regarding their attitudes and overall impressions towards AVs. Lexical choices have been shown to activate emotional information in memory and trigger valence-based reactions [39]. For instance, the use of positive valence trait words leads to more favorable evaluations compared to the use of negative valence trait words [40]. When individuals describe events, the vocabulary they employ often mirrors their emotional states [41]. This natural reflection of emotions through language is what makes lexical selection tasks a potent tool for unearthing the emotional components influencing AV perception and acceptance.

In the context of autonomous vehicles, the emotional lexicon of users can divulge prevalent attitudes. Do their word choices reflect optimism and reassurance or, conversely, fear and skepticism? Tracking how these choices change with exposure to autonomous vehicles can elucidate the interplay between emotion, cognition, and acceptance. For example, a shift towards more positive lexicon post-simulation experiences could indicate increasing comfort with the technology. Considering that emotional language serves as a valuable gauge of implicit attitudes, enriching explicit evaluations, current study aims to explore how lexical selection patterns reflecting underlying prevalent attitudes could be modulated as a consequence of a simulated exposure to a ride in an AV. Investigating the vocabulary associated with people’s perceptions of autonomous vehicles can unveil the depth at which emotional reactions steer the acceptance trajectory.

Finally, it is worth noting that the general public’s perception of AVs is shaped both by the intrinsic characteristics of the technology and by individual factors unique to each person. Ultimately, the degree of user adoption is largely contingent upon their idiosyncrasies. Extensive literature underscores that demographic variables substantially influence the perception and acceptance of AVs [8,42]. Notably, gender has emerged as a significant variable in numerous studies, revealing considerable disparities in attitudes towards autonomous technology [9]. Research consistently shows that men generally exhibit a more positive attitude and higher behavioral intention towards AVs than women [8]. Likewise, Schoettle and Sivak [22] found that 19% of male participants completely accepted AVs, compared to only 12.4% of females. Moreover, women expressed greater levels of concern, with 40% apprehensive about fully autonomous vehicles, versus 30% of men. Abraham et al. [43] substantiated these findings, revealing that 53% of males were comfortable allowing an autonomous vehicle full control, while only 40% of females echoed this sentiment. These gender differences in AV acceptance also extend to the perceived risk associated with the technology. Women generally express more concerns about the risks of AVs, scoring an average of 47.4% in the belief that autonomous vehicles will increase safety, compared to men’s average score of 60% [44]. Another significant demographic component is age. Younger individuals generally show more enthusiasm towards autonomous vehicle technology [42]. For instance, Piao et al. [45] conducted an online and telephone survey with over 400 participants and found that 62% of respondents aged between 18 and 34 were open to using AVs, compared to 56% of those over 65.

### 1.2. Research Rationale

In light of the crucial importance of public acceptance and perception for the successful integration of autonomous vehicles into society, our study is designed to examine how simulated experiences affect these attitudes. We aim to track variations in emotional positivity and activation levels after the simulation to gain a more detailed insight into participants’ emotional reactions. Accordingly, our primary research question asks: Does a positive simulated autonomous vehicle experience lead to improved perception and acceptance of these vehicles? Additionally, we explore two further questions: (1) Do adults initially exhibit a less favorable perception of autonomous vehicles compared to younger individuals? and (2) Do women possess comparatively lower levels of knowledge or trust in autonomous vehicles when compared to men?

## 2. Materials and Methods

### 2.1. Participants

For this research, we focused on enrolling participants from two different age categories: Younger (18 to 30 years) and Older (50 to 65 years). A total of 58 eligible individuals, with 32 of them being females, took part in the study. The recruitment process involved a targeted media outreach campaign. To be eligible, all participants had to meet specific criteria, which included possessing a valid driver’s license, having no relevant medical or psychological conditions, and demonstrating normal or corrected vision, hearing, and average cognitive functioning as evaluated by the Cognitive Assessment Battery—CAB^®^ (CogniFit Inc., San Francisco, CA, USA). Descriptive statistics of the participants are presented in Table 1.

The study was conducted in compliance with the principles of the Declaration of Helsinki and was approved by the Research Ethics Committee of Nebrija University, under the code UNNE-2023-0007. Prior to participation, all participants provided informed consent.

### 2.2. Materials and Procedure

We employed a questionnaire consisting of seven items, each with a 10-point Likert-scale response option. These items were grouped into three domains to evaluate participants’ knowledge and perceptions about autonomous vehicles. The first domain assessed participants’ understanding of autonomous vehicles with two questions: “How much do you know about autonomous vehicles?”, rated from 0 (I know nothing) to 10 (I know a lot), and “How much have you read or heard about autonomous vehicles?”, rated from 0 (nothing) to 10 (a lot). The aim was to gauge both awareness and informedness. The second domain explored participants’ perceptions of autonomous driving through three questions: “I have trusted the decisions made by the autonomous vehicle”, “I think the autonomous car has driven well”, and “I felt relaxed during the journey”, all rated from 0 (Totally disagree) to 10 (Totally agree). These questions were designed to measure trust, satisfaction, and comfort levels with autonomous driving. The third domain examined participants’ sense of safety during the drive, assessed by the question: “I felt the need or urge to take control of the steering wheel and/or pedals”, with responses ranging from 0 (Totally disagree) to 10 (Totally agree). To counter potential biases related to self-perceived driving skills, an additional question was included: “How well do you think you drive?”, ranging from 0 (Extremely poorly) to 10 (Extremely well). This question aimed to contextualize the responses within the framework of personal driving confidence. To ensure comparability and standardize responses across domains, given the varying number of items in each, the scores for each domain were calculated as the average of their respective items. All questions were mandatory, and there was no time limit set for responses.

To complement our understanding derived from the questionnaire, we implemented a lexical selection task comprising a set of 34 emotional normed words extracted from the Stadthagen–Gonzalez and colleagues database [46], forming pairs of oppositely charged words (positive and negative), along with neutral words as fillers. These words were characterized by standard arousal (mean = 5.48, standard deviation = 1.98) and valence values (mean = 5.35, standard deviation = 1.63). Participants were instructed to select the words that they linked with the term “autonomous vehicle”. These words were simultaneously displayed in a randomized order without any time constraints. The task was performed both before and after participants’ simulated drive experience. Detailed information about the words and their corresponding values can be found in Appendix A.

**Figure 1:**
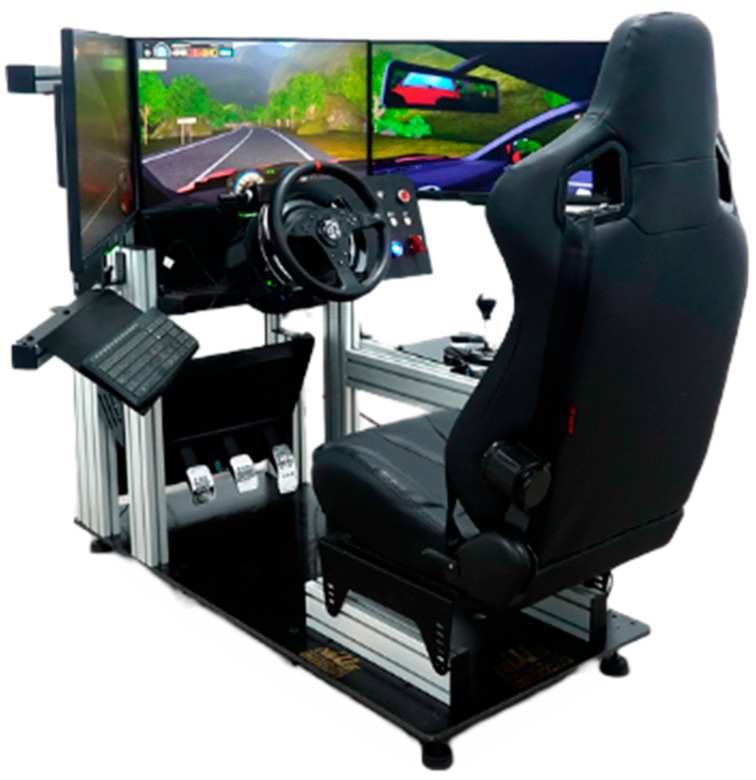
Simescar LITE driving simulator (SIMUMAK) used in the study.

The autonomous driving experience was conducted using a simulation that showcased a recorded video from the driver’s viewpoint. The simulation initiated with the vehicle parked in a designated parking area, then followed GPS directions traversing different road types, including an urban stretch, an interurban road, and a residential area where the car finally parked. The total distance traversed during the simulation was 4 km, with a duration of 12 min. The driving exercise took place within a virtual setting, making use of the Simescar LITE (SIMUMAK) cockpit equipped with vibration motion technology. This vibration system, albeit rudimentary, simulated the tremors associated with engine revolutions, bumps, or surface roughness, adding a tactile dimension to the experience. Three HP screens, providing a broad 180° field of view, were deployed to deliver an immersive visual experience (see Figure 1).

The experimental procedure set off with participants addressing the first domain items from the questionnaire and engaging in the lexical selection task. Following this, they were guided to the simulator to undergo the autonomous driving experience. Before setting the simulation in motion, participants were encouraged to visualize themselves in a self-driving car en route to a café, enhancing the psychological immersion in the scenario. Throughout the simulation, participants were instructed to maintain a passive role, with their primary objective being to observe the journey of the autonomous car within the virtual environment, devoid of any interaction requirement. Upon completion of the simulation, participants were encouraged to partake in the lexical selection task once more and to respond to the remaining items in the questionnaire. The entire session lasted approximately 30 min.

## 3. Results

The collected data were processed through RStudio [47] and subsequently analyzed using jamovi [48]. The threshold for statistical significance was established at *p* < 0.05, and partial eta squared was computed to measure the magnitude of observed effects.

The data derived from the questionnaire were subjected to Analysis of Covariance (ANCOVA) with Knowledge Pertaining to Autonomous Vehicles, Perception of Autonomous Driving, and Perceived Safety During Driving as the defined dependent variables. Age Group and Gender were introduced as fixed factors, with the participant’s Self-perceived Driving Ability factored in as a covariate.

As it pertains to the lexical association task, a Repeated Measures Analysis of Variance (ANOVA) was employed. This method facilitated an evaluation of the impact of the simulated autonomous driving experience factoring in Age Group and Gender (between-subjects) and Test Moment (within-subjects) on Mean Number of Selected Words, Mean Valence, and Mean Arousal.

### 3.1. Autonomous Vehicles: Divergences in Knowledge, Perception, and Safety

**Figure 2:**
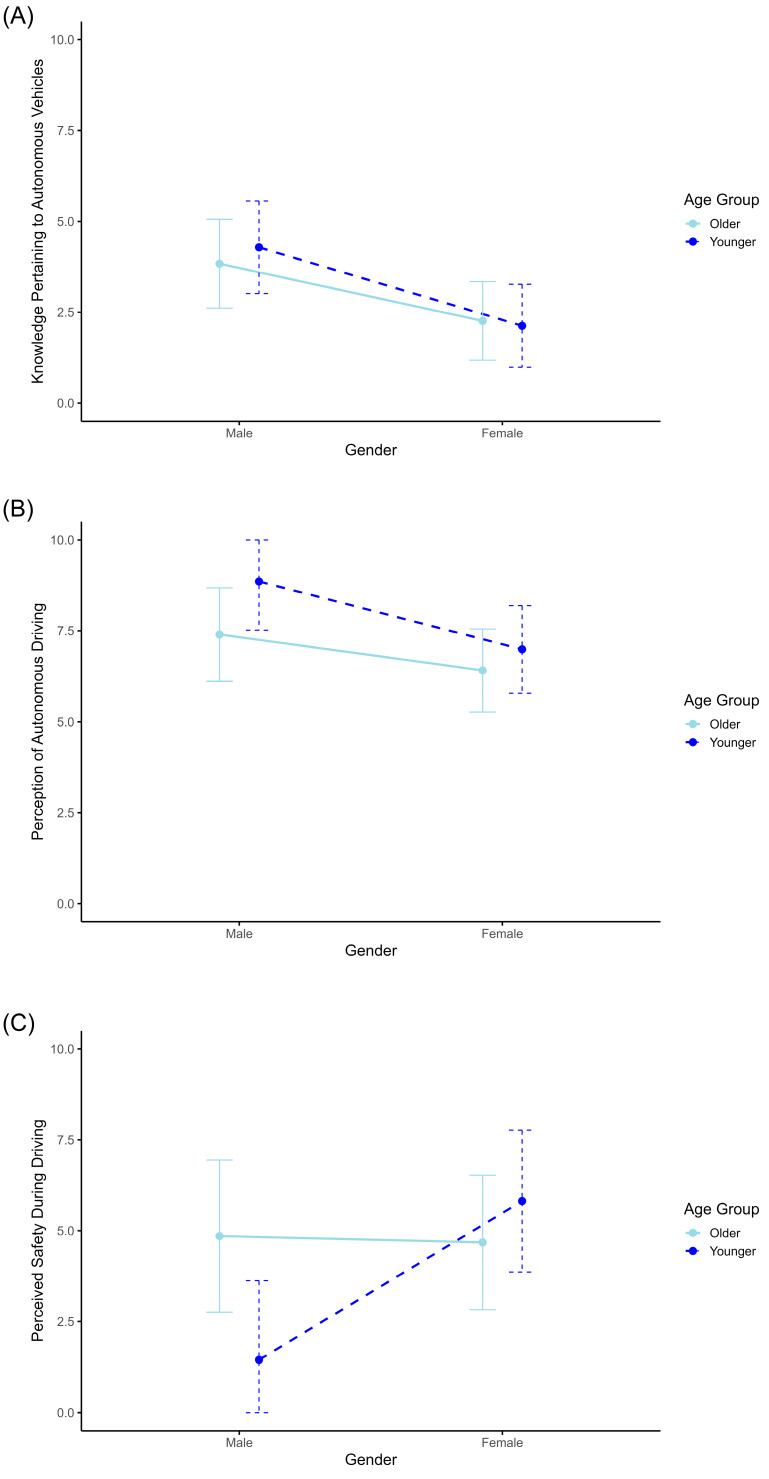
Gender and age group variations in autonomous vehicle knowledge, perception, and perceived safety. The scores represent adjusted estimated marginal means for gender and age group, assessing Knowledge Pertaining to Autonomous Vehicles (**A**), Perception of Autonomous Driving (**B**), and Perceived Safety During Driving (**C**). Error bars depict 95% confidence intervals.

The preliminary analyses of our investigation involved an ANCOVA, wherein Knowledge Pertaining to Autonomous Vehicles functioned as the dependent variable. The findings unveiled a significant main effect of Gender (F(1, 53) = 9.76, *p* = 0.003, ∂η^2^ = 0.156), indicating that female participants possessed less knowledge about autonomous vehicles compared to their male counterparts (Figure 2A). Age Group did not yield a significant effect. Importantly, Self-perceived Driving Ability as a covariate exerted a significant influence on the dependent variable (F(1, 53) = 4.45, *p* = 0.04, ∂η^2^ = 0.077). Namely, participants who considered themselves to be very skilled drivers reported greater knowledge about autonomous vehicles.

The subsequent analysis revealed a significant effect of Gender on the Perception of Autonomous Driving (F(1, 53) = 5.15, *p* = 0.027, ∂η^2^ = 0.089). Female participants reported feeling more uncomfortable during autonomous driving compared to males (Figure 2B). No significant effects were found for Age Group or for the interaction between Age Group and Gender. Additionally, when considering Self-perceived Driving Ability as a covariate, it did not show significant interactions with Age Group or Gender.

A third ANCOVA analysis was conducted with Perceived Safety During Driving as the dependent variable. This variable’s scores are directly tied to the item “I felt the need or urge to take control of the steering wheel and/or pedals,” representing an inverse measure of perceived safety. Results showed a significant main effect of gender (F(1, 53) = 4.21, *p* = 0.045, ∂η^2^ = 0.074), implying that female participants experienced a higher need to take control of the vehicle, which translates to a lower level of perceived safety during autonomous driving compared to male participants. No significant effects for Age Group were identified. Nevertheless, a significant interaction effect was found between Gender and Age Group (F(1, 53) = 5.03, *p* = 0.029, ∂η^2^ = 0.087). Younger male participants reported lower scores on the need to take control, suggesting a higher level of confidence with autonomous driving (Figure 2C).

### 3.2. Emotional Responses to Autonomous Vehicle Simulation Experience

**Figure 3:**
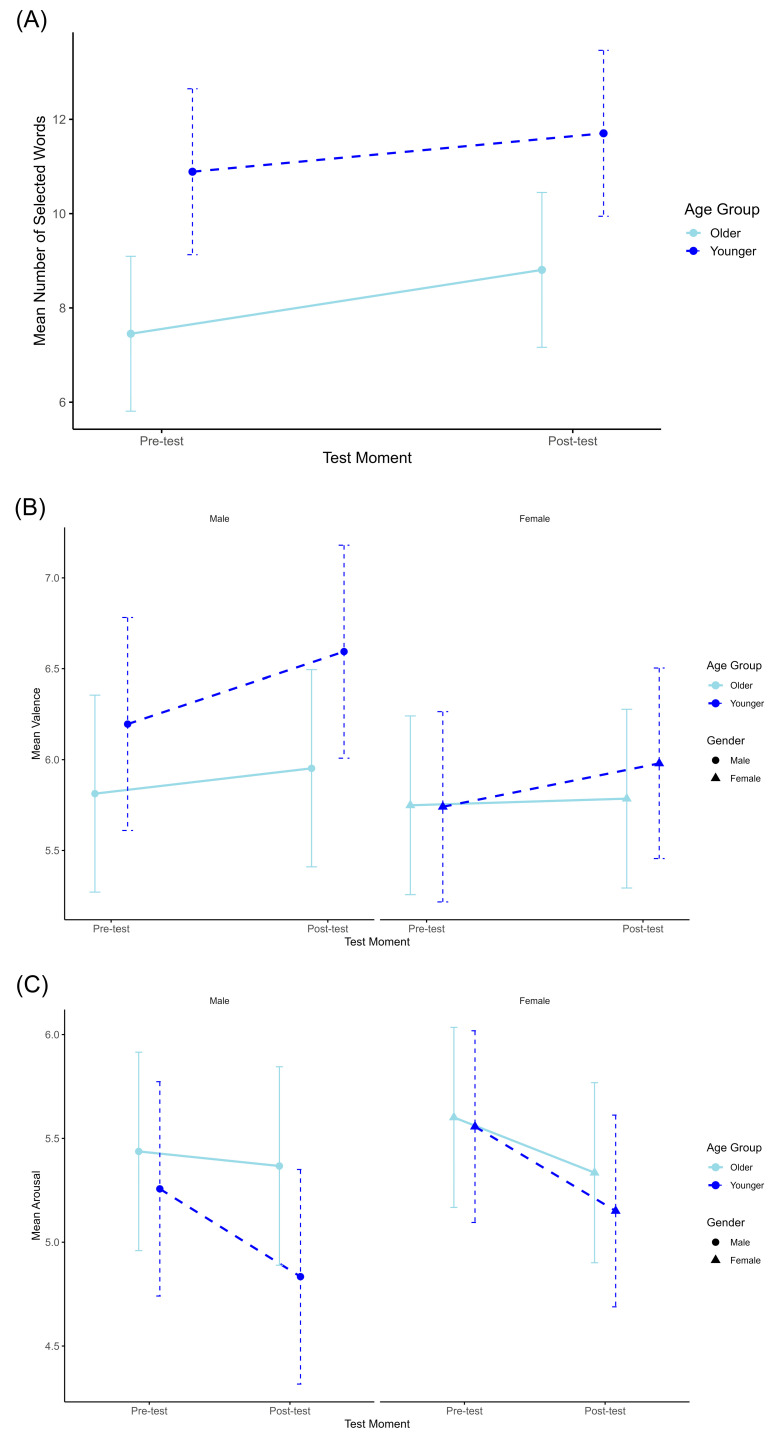
Gender and age group variations in linguistic selection, emotional valence, and arousal levels before and after simulated autonomous vehicle experience. The scores in this figure represent adjusted estimated marginal means across gender and age groups, evaluating the linguistic and emotional responses to the autonomous vehicle simulation experience. (**A**) Presents the comparison of the mean number of selected words between pre- and post-test evaluations. (**B**,**C**) Exhibits mean valence and arousal ratings, respectively, for pre- and post-test evaluations by gender and age group. Error bars represent 95% confidence intervals, reflecting the variance in participants’ responses.

In the second phase of our analysis, an initial ANOVA with the Mean Number of Selected Words as the dependent variable was conducted. This compared the selected word counts from the pre-test and post-test of the simulated autonomous driving experience. Age Group and Gender were treated as between-subjects factors. Results indicated a significant difference (F(1, 54) = 6.20, *p* = 0.016, ∂η^2^ = 0.103), with an increased selection of words post-experience (Figure 3A). Furthermore, the effect of Age Group was also significant (F(1, 54) = 7.20, *p* = 0.01, ∂η^2^ = 0.118), with younger participants tending to select a higher number of words. No gender differences were found.

Subsequent analysis evaluated the Mean Valence of the selected words as the dependent variable, considering the Test Moment as a within-subject factor and Age Group and Gender as between-subjects factors. The results showed a slight trend towards significance (F(1, 54) = 2.82, *p* = 0.099, ∂η^2^ = 0.05), with post-test words tending to exhibit higher valence (Figure 3B). This suggests a greater association of positively charged words with the autonomous driving experience among participants. Even though this trend was more pronounced among male and younger participants, the differences based on Gender and Age Group were not statistically significant.

Finally, we conducted a repeated measures ANOVA for the Mean Arousal, considering the Test Moment as a within-subjects factor and Age Group and Gender as between-subjects factors. The results showed a significant effect (F(1, 54) = 6.42, *p* = 0.014, ∂η^2^ = 0.106), characterized by a decline in average arousal levels post-simulation (Figure 3C). This implies that participants felt more tranquil and relaxed following the experience. No significant differences based on Gender or Age Group were observed.

## 4. Discussion

Despite the demonstrated advancements and effectiveness of autonomous vehicles (AVs), societal acceptance and adoption of this technology remain somewhat hesitant. Notably, a gap exists between the technical capabilities of AVs and their widespread acceptance, which is influenced by numerous factors including knowledge, perception, safety concerns, and demographic characteristics. Understanding public perception towards AVs is crucial for laying the groundwork for their successful societal integration. The current study aims to evaluate the perception of the Spanish population towards AVs and investigate the extent to which exposure to this technology could shape our perceptions. To accomplish this, we employed a driving simulation approach that offered participants a firsthand experience of AVs in a safe and controlled setting.

In the course of our study, we recruited participants through a targeted media outreach campaign, ensuring balance in gender representation and a diverse age range. Utilizing a questionnaire and lexical selection tasks, we explored participants’ knowledge and attitudes towards AVs both before and after the immersive simulation experience. This method allowed us to gauge changes in participants’ perceptions, considering both cognitive understanding and emotional responses to AVs, enriching our analysis with a multi-dimensional perspective on public acceptance.

Our research, echoing findings from broader studies, reveals that female participants generally reported lower levels of knowledge about AVs compared to their male counterparts. This trend is not isolated but rather a widespread observation seen across various countries [21,22]. Additionally, our findings show that participants who rated themselves as skilled drivers tended to have a more profound understanding of AVs. This reflects a well-documented phenomenon, where individuals’ self-assessment of their abilities correlates strongly with their actual performance [49]. In line with preceding reports [50], our study has identified a notable gender difference in the perception of autonomous driving, indicating distinct attitudes and comfort levels between male and female participants. Specifically, it was observed that women generally reported feeling less comfortable with autonomous driving compared to their male counterparts. This variation in comfort levels can be understood through the lens of group affiliations and perceptions. Typically, human drivers are perceived as a more relatable and diverse group (the ‘in-group’), whereas autonomous vehicles are often seen as a distinct, less familiar ‘out-group’ [51]. Machines, particularly those belonging to the same category, like autonomous vehicles, are commonly perceived as more homogenous compared to the diverse nature of humans. This perception is closely linked to one’s familiarity and knowledge about a particular group. In this context, those with lower levels of knowledge about technology, especially autonomous driving, are more likely to perceive these vehicles as an ‘out-group’. The literature, as well as our study, reveals that females and older individuals consistently report having lesser knowledge about technology compared to males and younger people. This disparity in knowledge and familiarity likely contributes to the heightened discomfort among these demographics. Consequently, their perception of autonomous vehicles as an ‘out-group’ is intensified, leading to increased apprehension and a stronger desire for control when interacting with these systems. These dynamics shed light on the deeper social and psychological mechanisms that influence attitudes towards emerging technologies, highlighting the importance that knowledge and familiarity play in shaping these perceptions.

Concerning age-related trends, our data show a slight inclination where older participants might perceive autonomous driving more negatively than younger ones, yet this trend did not reach statistical significance. This contrast from prior research findings, which found such differences [52,53,54], may be linked to the heightened exposure of urban adults to recent technological advancements via media and social networks. Residing in a large city generally results in more frequent encounters with new technologies [55,56], which could contribute to the relatively positive perceptions observed among our city-dwelling participants. We also observed gender differences regarding perceived safety. Women, regardless of age, expressed a moderate level (5 out of 10) of need to take control of the vehicle at some point during the journey. On the other hand, older men also felt insecure about autonomous driving, reporting similar levels of need for control as women. However, a distinct pattern emerged among young men, who felt calm and secure during the driving experience, registering a notably lower mean of 1.42 out of 10 on the need for control scale. This moderate perceived safety among participants can be contextualized within the framework of social psychology and human judgment. Findings from Zhang et al. [57] showed that participants are more likely to assign blame to autonomous systems than to human drivers in identical traffic incident scenarios. This inclination increases with the severity of the incident’s outcome. Such a bias reflects a cognitive predisposition to distrust autonomous vehicles, possibly due to a perceived lack of nuanced judgment and associative reasoning that humans are believed to possess.

Taken together, the gender effect remains significant across all three domains: knowledge, perception, and safety. Generally, women tend to have less knowledge about autonomous vehicles, perceiving them as riskier and less comfortable compared to men. These findings align with previous studies on attitudes towards autonomous vehicles and willingness to use them [58,59,60], and could be explained by gender differences in personality traits such as sensation-seeking [61]. Regarding the critical question of the need to take control, people, in general, expressed moderate discomfort and a moderate level of need for control. An exceptional case is observed among young men, who felt more comfortable with autonomous vehicles and have a lower need to take control, suggesting that the perception of safety during autonomous driving may be influenced by the complex interplay between gender and age.

To further investigate the direct experience with autonomous vehicle simulation and its potential to shape participants’ attitudes, we included a lexical selection task where participants were asked to select from a standardized list of words those that they associated with “autonomous vehicles”. This task aimed to explore whether drivers incidentally acquire more knowledge after being exposed to an autonomous vehicle. The results revealed that, on average, younger individuals provided a higher number of words, indicating a greater knowledge of autonomous vehicles compared to older individuals. Furthermore, participants selected more words after engaging with the simulator, suggesting that the experience provided them with additional information about this technology. Overall, our findings suggest that having direct experience with an autonomous vehicle enables individuals to provide more information, and in general, younger individuals possess a greater knowledge of autonomous vehicles compared to older individuals.

After addressing the previous question, the next step was to determine the emotional bias or activation level of the information acquired. To assess this, we analyzed the arousal and valence values associated with the words selected by the participants before and after experiencing the simulated autonomous driving conditions. As expected, due to the tight relationship between acceptance and emotional processes [62], our results revealed a significant main effect of arousal, with a significant decrease in scores when comparing the average before and after the simulated autonomous driving experience. This finding contrasts with the observations in the Payre et al. [63] study, where no significant difference in fully automated driving acceptability was noted before and after interaction with the simulated system. However, an important aspect to consider is that their questionnaire compared automated driving with manual driving. As a result, the increase in acceptance of automated driving may not be strong enough to surpass confidence in manual driving. Despite this, Payre et al. [63] did find that simulation experience increased interest in delegating control to the autonomous system in situations where the driver was impaired, such as tiredness or alcohol consumption. This nuanced view from their study provides a broader context to our findings. The change we observed indicates a reduction in the intensity of the experience after going through the simulator. The selected words were calmer, positioning participants in a better state to understand and face autonomous vehicles, minimizing potential negative impacts. This shift aligns with the Risk-as-Feelings hypothesis [64], which emphasizes how visceral impressions of risk differ from analytical evaluations, leading to a mitigation of initial anxiety and fears stemming from unfamiliarity. This, in turn, gives rise to more positive emotions, which subsequently predict greater acceptance. Regarding valence, although the scores were higher, implying a more positive emotive response, the differences were not statistically significant.

In summary, the results indicate that, regardless of gender and age, the experience with autonomous vehicles influences the quantity and quality of information, resulting in greater knowledge, more positive emotions, and a sense of calmness. It is noteworthy that women consistently exhibited lower levels of information and perceived safety, as well as negative valence and higher arousal. Similarly, although not statistically significant, the average scores followed a similar trend for older individuals compared to younger ones.

### 4.1. Practical Implications

The successful adoption of autonomous vehicles is contingent upon building public trust, increasing exposure, and addressing safety and control concerns. Our research sheds light on practical implications that can effectively promote acceptance and positive perceptions of autonomous vehicles.

Exposure to autonomous vehicles through simulation significantly enhances understanding and elicits a more positive and calm emotional response across diverse genders and age groups. In addition, the experience of simulation leads to reduced arousal levels, indicating a greater sense of calmness and relaxation. Consequently, immersive simulations play a crucial role in alleviating apprehensions and fostering comfort with autonomous vehicle technology. To promote social acceptance and familiarity with autonomous vehicles, it is imperative to offer initiatives, such as virtual simulators or safe test drive environments. By dismantling social barriers and bringing this technology closer to the public, we can harness the numerous societal benefits it offers. It is important to note, that while our study emphasizes the benefits of simulation experiences in improving societal perceptions, we do not specifically advocate for mandatory simulation training for operating autonomous vehicles. Instead, our research suggests the broader implementation of initiatives that increase public exposure and familiarity with these vehicles.

Public institutions that provide secure environments for test drives or virtual simulators are essential in advancing the social acceptance and widespread adoption of autonomous vehicles. Drawing on the findings of Charness et al. [65], and supported by the results of Lee et al. [66], which showed how the public’s trust and intention to adopt AVs can be increased through positive information on social and traditional media, our research underscores the significance of increasing familiarity and exposure to autonomous vehicle technology to alleviate concerns and cultivate favorable attitudes towards vehicle automation.

Consequently, it is critical to design strategies that bolster public confidence in autonomous vehicles, encompassing measures to augment exposure, raise awareness, and address specific safety and control concerns. These strategies should include, but not be limited to, the use of immersive technologies, such as simulators. They should also encompass real-world experiences, such as designated routes or areas for autonomous vehicles in urban settings, as proposed in our study. Moreover, it is essential to tailor these strategies to cater to different demographic groups. Notable variations in the acceptance of autonomous vehicles based on gender and age underscore the need to account for these differences in the design, marketing, and policy decisions pertaining to autonomous vehicles. By acknowledging gender-specific comfort and trust issues, targeted initiatives can be developed to address the concerns of women explicitly.

### 4.2. Limitations and Future Research Directions

While our study provides valuable insights into public acceptance and perceptions of autonomous vehicles, it is essential to acknowledge several limitations. Firstly, we employed a simulation to replicate the autonomous driving experience, which, while immersive and designed to simulate aspects of an autonomous vehicle experience, such as visual perspective and tactile feedback, still falls short of fully capturing the unpredictable and complex dynamics of real-world driving scenarios. Consequently, participants’ responses and attitudes may differ when faced with actual on-road conditions, considering other road users and varying environmental factors. Secondly, our study predominantly focused on gender and age differences, overlooking other potentially significant demographic and socio-economic factors such as income, education, or geographic location. Exploring these factors could offer additional insights into public perception and acceptance of autonomous vehicles. Thirdly, the use of a narrative scenario, where participants imagined themselves in a real-life situation (e.g., taking a taxi to a café), was intended to enhance the realism of the simulation, yet this approach has its limitations in replicating the full breadth of experiences and interactions with a real autonomous vehicle. Finally, while our sample size was sufficient for our analysis, it remained relatively small. A larger and more diverse sample would yield a more comprehensive understanding of the population’s perspective on autonomous vehicles.

Based on these limitations, there are several promising avenues for future research. One important direction would be to examine the acceptance and perception among individuals who cannot or do not currently drive, including those with motor limitations or individuals who are beyond the legal driving age. Understanding how these populations perceive and accept autonomous vehicles is of great significance, as they stand to benefit greatly from this technology. In fact, the elderly and individuals with disabilities are often assumed to be early adopters due to the increased accessibility offered by autonomous vehicles [9].

Furthermore, it would be valuable to replicate and expand upon our findings in real-world conditions using actual autonomous vehicles. This would help ascertain whether the positive shifts in perception and decrease in arousal observed in the simulator study translate to real-life experiences. By conducting research in real-world settings, we can gain a more comprehensive understanding of how autonomous vehicles are perceived and accepted in practical situations.

## 5. Conclusions

This study aimed to explore how individuals experience autonomous driving and how interacting with an autonomous vehicle may influence their attitudes and acceptance towards this technology. Through the use of a driving simulator, we investigated the emotional reactions of both young and adult participants to autonomous driving in a virtual environment. The findings suggest that individual differences are important to consider when designing social awareness programs aimed at promoting the acceptance of autonomous driving technology. These insights can inform the development of effective strategies for promoting the widespread adoption of autonomous vehicles.

## Figures and Tables

**Table 1:** Descriptive statistics of the participants.

	Age Group	Gender	N	Mean	SD	Min.	Max.
Age	Younger	Male	12	23.3	2.39	20	28
	Younger	Female	15	23.6	2.20	21	27
	Older	Male	14	57.6	4.27	51	65
	Older	Female	17	56.2	3.30	51	63

Note. N = number of participants, SD = standard deviation, Min = minimum value, Max = maximum value.

## Data Availability

The data that support the findings of this study are available upon request from the corresponding author.

## Appendix A. Word List and Standardized Arousal and Valence Values

**Word (Spanish)**	**Word (English)**	**Valence**	**Arousal**
agradable	pleasant	8	2.8
angustia	anguish	1.95	7.25
aprensión	apprehensiveness	3.75	6
autonomía	autonomy	6.85	4.4
certeza	certainty	7.05	4.25
coherencia	coherence	7.5	4.1
complejidad	complexity	4.5	6.2
confianza	confidence	8.18	2.85
electricidad	electricity	5.35	6.1
error	error	2.9	6.5
fiabilidad	reliability	6.65	4.75
firmeza	firmness	5.95	5.4
funcionalidad	functionality	6.2	5
futuro	future	6.35	6.35
impreciso	imprecise	4.15	6
incertidumbre	uncertainty	3.55	7.2
independencia	independence	6.8	5.05
inestabilidad	instability	2.25	6.8
innovación	innovation	7.05	5.5
inquietud	concern	3.5	7.1
inteligencia	intelligence	7.85	5.65
mecánico	mechanical	4.95	5.2
miedo	fear	2	8
modernidad	modernity	6.1	5.4
movilidad	mobility	6.9	5.35
peligro	danger	2.93	8
preocupación	worry	2.35	7.3
protección	protection	7.2	4.55
relajación	relaxation	7.25	1.5
riesgo	risk	4.6	7.1
seguridad	security	6.95	2.58
sostenibilidad	sustainability	6.25	4.45
tecnología	technology	6.2	5.4
tranquilidad	tranquility	7.8	1.75
utilidad	utility	7.3	4.75
vulnerabilidad	vulnerability	2.3	6.15
